# Supplementary material for: Two distinct immunopathological profiles in autopsy lungs of COVID-19
Source: Nat Commun. 2020 Oct 8;11:5086. doi: 10.1038/s41467-020-18854-2 (PMC7546638; doi:10.1038/s41467-020-18854-2)
Supplement: Supplementary file 1 — Supplementary Information [file 41467_2020_18854_MOESM1_ESM.pdf]

# Two distinct immunopathological profiles in autopsy lungs of COVID-19

## Supplementary Information

Supplementary Table 1. Clinicopathological data of $ISG^{high}$ and $ISG^{low}$ patients.

| **Case No.** | **No. of OIRRA biopsies** | **Age** | **Sex** | **BMI** | **Hospitalisation period (days)** | **Comorbidities** | | **Therapy** | **Cause of death** | **Intubation** (invasive) | **Thromboembolic events in lung** | **Disseminated intravascular coagulation** |
|---|---|---|---|---|---|---|---|---|---|---|---|---|
| n=16 | n=34 | | | | | **Cardiovascular** (with risk factors) | **Pulmonary** (with risk factors) | | | Days of Intubation | Pulmonary microthrombi, embolism | |
| **C1** | 2 | 67 | F | 35 | 9 | HT, obesity, atherosclerosis | 0 | HCQ, AB | COVID-19 associated respiratory failure | 0 | **1** | 0 |
| **C5** | 3 | 66 | M | 29 | 9 | HT, CAD | smoker | L/R, HCQ, AB, Remdesivir, antifungal | COVID-19 associated respiratory failure, multiorgan failure | **12** | 0 | 0 |
| **C13** | 1 | 53 | M | 59 | 8 | HT, obesity, DM | sleep apnea, long term oxygen therapy (h/o ARDS with respiratory failure) | HCQ, AB | COVID-19 associated respiratory failure | **8** | **1** | 0 |
| **C16** | 2 | 61 | F | 41 | 9 | HT, obesity, DM, CAD | 0 | AB | COVID-19 associated respiratory failure | 0 | 0 | 0 |
| **C17** | 2 | 72 | M | 25 | 12 | HT, DM, cardiac arrhythmia | smoker, COPD, sleep apnea | AB | COVID-19 associated respiratory failure | 0 | **1** | **1** |
| **C20** | 1 | 71 | M | 36 | 4 | HT, obesity, dyslipidemia, CAD | smoker, COPD | HCQ, AB | COVID-19 associated respiratory failure | 0 | 0 | **1** |
| **C21** | 3 | 96 | M | 25 | 13 | HT, sick sinus syndrome with pacemaker implantation, CAD | 0 | HCQ, AB | COVID-19 associated respiratory failure | **4** | 0 | **1** |
| **C4** | 2 | 77 | M | 44 | 3 | HT, obesity, DM, dyslipidemia, aortic valve reconstruction, atrial fibrillation, CAD | smoker, sleep apnea | L/R, HCQ, AB | COVID-19 associated respiratory failure | 0 | 0 | 0 |
| **C6** | 3 | 74 | M | 27 | 3 | HT, DM, CAD | smoker, COPD | L/R, HCQ | COVID-19 associated respiratory failure | 0 | 0 | 0 |
| **C7** | 3 | 81 | F | 26 | 4 | HT, CAD, peripheral artery disease | smoker, COPD | L/R, HCQ | COVID-19 associated respiratory failure | 0 | 0 | 0 |
| **C9** | 3 | 88 | M | 28 | 2 | HT, right heart failure, coronary and hypertensive heart disease, atrial fibrillation | 0 | L/R, HCQ | COVID-19 associated respiratory failure | 0 | 0 | 0 |
| **C12** | 3 | 75 | M | 27 | 3 | HT, DM, dyslipidemia, CAD | sleep apnea | HCQ, AB | COVID-19 associated respiratory failure | 0 | 0 | 0 |
| **C15** | 1 | 89 | M | 26 | 5 | HT, DM, dyslipidemia, CAD, valvular, hypertensive and rhythmogenic heart disease, AV-block III° | ex-smoker | AB | COVID-19 associated respiratory failure | 0 | 0 | 0 |
| **C19** | 1 | 65 | M | 26 | 7 | HT, DM, CAD | ex-smoker, COPD, sleep apnea | AB | COVID-19 associated respiratory failure | 0 | 0 | 0 |
| **C3** | 1 | 95 | M | 23 | 3 | HT, CAD, myocardial infarction, aortic stenosis | 0 | AB | COVID-19 associated respiratory failure with bacterial superinfection | 0 | **1** | 0 |
| **C8** | 3 | 71 | M | 25 | 0 | HT, CAD, valvular heart disease, peripheral arterial disease, infrarenal aortic aneurysm | 0 | best supportive care | COVID-19 associated respiratory failure with bacterial superinfection and multiorgan failure | 0 | **1** | 0 |

HT = Hypertension, CAD = Coronary artery disease, DM = Diabetes Mellitus, COPD = Chronic obstructive pulmonary disease, HCQ = Hydroxychloroquine, AB = Antibiotics, L/R = Lopinavir/Ritonavir

Supplementary Table 1. Clinicopathological data of $ISG^{high}$ and $ISG^{low}$ patients.
$ISG^{high}$ patients, red; $ISG^{low}$ patients, blue. The Table shows the relevant parameters for all patients, including the cause of death that was respiratory failure, in two out of 16 patients with consequent multi-organ failure. Study patients with unambiguous sample segregation in either Cluster 1 or 2 were assigned the corresponding ISG activation label $ISG^{high}$ and $ISG^{low}$, respectively. Two patients (C3 and C8) could not be unambiguously assigned to one or the other ISG pattern:

C3: Only one tissue block was available for molecular characterization. This sample was part of cluster 3 = normal. Therefore, patient C3 was not assigned to either of the groups ($ISG^{high}$ or $ISG^{low}$).

C8: Three tissue blocks were available for molecular characterization. These three samples all grouped in different clusters. Therefore, patient C8 was not assigned to either of the groups ($ISG^{high}$ or $ISG^{low}$).

Supplementary Table 2. OIRRA gene list

| | | | | | | | |
|---|---|---|---|---|---|---|---|
| ABCF1 | CCL5 | CD40LG | CTAG2 | FASLG | HIF1A | IFIT1 | IL3RA |
| ADGRE5 | CCNB2 | CD44 | CTLA4 | FCER1G | HLA-A | IFIT2 | IL4 |
| ADORA2A | CCR1 | CD47 | CTSS | FCGR1A | HLA-B | IFIT3 | IL6 |
| AIF1 | CCR2 | CD48 | CX3CL1 | FCGR2B | HLA-C | IFITM1 | IL7 |
| AKT1 | CCR4 | CD52 | CX3CR1 | FCGR3A | HLA-DMA | IFITM2 | IL7R |
| ALOX15B | CCR5 | CD53 | CX3CR1 | FCGR3B | HLA-DMB | IFNA17 | IRF1 |
| ARG1 | CCR6 | CD6 | CX3CR1 | FCRLA | HLA-DOA | IFNB1 | IRF4 |
| AXL | CCR7 | CD63 | CX3CR1 | FOXM1 | HLA-DOB | IFNG | IRF9 |
| B3GAT1 | CD14 | CD68 | CXCL1 | FOXO1 | HLA-DPA1 | IGF1R | IRS1 |
| BAGE | CD160 | CD69 | CXCL10 | FOXP3 | HLA-DPB1 | IGSF6 | ISG15 |
| BATF | CD163 | CD70 | CXCL11 | FUT4 | HLA-DQA1 | IKZF1 | ISG20 |
| BCL2 | CD19 | CD74 | CXCL13 | FYB | HLA-DQA2 | IKZF2 | ITGA1 |
| BCL2L11 | CD1C | CD79A | CXCL8 | G6PD | HLA-DQB2 | IKZF3 | ITGAE |
| BCL6 | CD1D | CD79B | CXCL9 | GADD45GIP1 | HLA-DRA | IKZF4 | ITGAL |
| BRCA1 | CD2 | CD80 | CXCR2 | GAGE1 | HLA-DRB1 | IL10 | ITGAM |
| BRCA2 | CD209 | CD83 | CXCR3 | GAGE10 | HLA-E | IL10RA | ITGAX |
| BST2 | CD22 | CD86 | CXCR4 | GAGE12J | HLA-F | IL12A | ITGB1 |
| BTLA | CD226 | CD8A | CXCR5 | GAGE13 | HLA-F-AS1 | IL12B | ITGB2 |
| BUB1 | CD244 | CD8B | CXCR6 | GAGE2C | HLA-G | IL13 | ITGB7 |
| C10orf54 | CD247 | CDK1 | CYBB | GATA3 | HMBS | IL15 | ITK |
| C1QA | CD27 | CDKN2A | DDX58 | GBP1 | ICAM1 | IL17A | JAML |
| C1QB | CD274 | CDKN3 | DGAT2 | GNLY | ICOS | IL17F | JCHAIN |
| CA4 | CD276 | CEACAM1 | DMBT1 | GPR18 | ICOSLG | IL18 | KIAA0101 |
| CBLB | CD28 | CEACAM8 | EBI3 | GRAP2 | ID2 | IL1A | KIR2DL1 |
| CCL17 | CD33 | CIITA | EFNA4 | GUSB | ID3 | IL1B | KIR2DL2 |
| CCL18 | CD37 | CLEC4C | EGFR | GZMA | IDO1 | IL2 | KIR2DL3 |
| CCL2 | CD38 | CMKLR1 | EGR2 | GZMB | IDO2 | IL21 | KLF2 |
| CCL20 | CD3D | CORO1A | EGR3 | GZMH | IFI27 | IL22 | KLRB1 |
| CCL21 | CD3E | CRTAM | EIF2AK2 | GZMK | IFI35 | IL23A | KLRD1 |
| CCL22 | CD3G | CSF1R | ENTPD1 | HAVCR2 | IFI44L | IL2RA | KLRF1 |
| CCL3 | CD4 | CSF2RB | EOMES | HERC6 | IFI6 | IL2RB | KLRG1 |
| CCL4 | CD40 | CTAG1B | FAS | HGF | IFIH1 | IL2RG | KLRK1 |

| | | | | |
|---|---|---|---|---|
| KREMEN1 | MLANA | POLR2A | SSX2 | TNFSF9 |
| KRT5 | MMP2 | POU2AF1 | STAT1 | TOP2A |
| KRT7 | MMP9 | PRDM1 | STAT3 | TP63 |
| LAG3 | MPO | PRF1 | STAT4 | TRIM29 |
| LAMP1 | MRC1 | PSMB9 | STAT5A | TUBB |
| LAMP3 | MS4A1 | PTEN | STAT6 | TWIST1 |
| LAPTM5 | MTOR | PTGS2 | TAGAP | TYROBP |
| LCK | MX1 | PTK7 | TAP1 | VCAM1 |
| LCN2 | MYC | PTPN11 | TARP | VEGFA |
| LEXM | NCAM1 | PTPN6 | TBP | VTCN1 |
| LILRB1 | NCF1 | PTPN7 | TBX21 | XAGE1B |
| LILRB2 | NCR1 | PTPRC | TCF7 | ZAP70 |
| LMNA | NCR3 | PTPRCAP | TDO2 | ZBTB46 |
| LRG1 | NECTIN2 | PVR | TFRC | ZEB1 |
| LRP1 | NFATC1 | PYGL | TGFB1 | |
| LST1 | NFKBIA | RB1 | TIGIT | |
| LY9 | NKG7 | RORC | TLR3 | |
| LYZ | NOS2 | RPS6 | TLR7 | |
| M6PR | NOTCH3 | S100A8 | TLR8 | |
| MAD2L1 | NRP1 | S100A9 | TLR9 | |
| MADCAM1 | NT5E | SAMHD1 | TNF | |
| MAGEA1 | NTN3 | SDHA | TNFAIP8 | |
| MAGEA10 | OAS1 | SELL | TNFRSF14 | |
| MAGEA12 | OAS2 | SH2D1A | TNFRSF17 | |
| MAGEA3 | OAS3 | SH2D1B | TNFRSF18 | |
| MAGEA4 | PDCD1 | SIT1 | TNFRSF4 | |
| MAGEC2 | PDCD1LG2 | SKAP2 | TNFRSF9 | |
| MAPK1 | PECAM1 | SLAMF7 | TNFSF10 | |
| MAPK14 | PGF | SLAMF8 | TNFSF13B | |
| MELK | PIK3CA | SNAI1 | TNFSF14 | |
| MIF | PIK3CD | SNAI2 | TNFSF18 | |
| MKI67 | PMEL | SRGN | TNFSF4 | |

Supplementary Table 3. Differentially expressed genes, COVID-19 versus controls

| ID | logFC | logCPM | PValue | FDR |
|---|---|---|---|---|
| DMBT1_64696575 | 5.086440047 | 9.507465672 | 8.27E-12 | 3.29E-09 |
| TDO2_55162 | 3.60666041 | 9.53520997 | 3.39E-11 | 6.12E-09 |
| IFI6_47156 | 3.778639284 | 14.24783272 | 4.61E-11 | 6.12E-09 |
| KIAA0101_319426 | 3.146999681 | 8.549063739 | 8.30E-11 | 8.26E-09 |
| IGF1R_12291338 | -1.346908853 | 10.50777116 | 1.63E-10 | 1.29E-08 |
| MELK_300401 | 2.899390615 | 7.833266318 | 9.68E-10 | 6.29E-08 |
| BUB1_701803 | 2.429206201 | 8.360010662 | 1.11E-09 | 6.29E-08 |
| ISG15_66173 | 4.276622596 | 13.15626013 | 1.97E-09 | 9.78E-08 |
| IFI27_37143 | 1.911305772 | 12.5471898 | 3.00E-09 | 1.33E-07 |
| PSMB9_384491 | 1.385731366 | 10.9106894 | 6.49E-09 | 2.58E-07 |
| OAS3_667776 | 2.754666249 | 9.890613941 | 9.75E-09 | 3.53E-07 |
| CDK1_837939 | 2.262910126 | 9.211945164 | 1.08E-08 | 3.59E-07 |
| HLA-G_483585 | 4.030812546 | 6.375748944 | 1.47E-08 | 4.35E-07 |
| SLAMF8_9071016 | 2.293400189 | 9.725983998 | 1.53E-08 | 4.35E-07 |
| MLANA_159265 | -5.75771358 | 0.738276085 | 1.83E-08 | 4.86E-07 |
| CDKN3_434534 | 1.939803243 | 8.894315977 | 2.32E-08 | 5.46E-07 |
| OAS1_757865 | 2.607400402 | 11.38534225 | 2.33E-08 | 5.46E-07 |
| JAML_78188 | -1.759772394 | 7.103911839 | 6.73E-08 | 1.49E-06 |
| IRS1_37283828 | -1.354465089 | 10.24560172 | 7.98E-08 | 1.62E-06 |
| OAS2_15981707 | 2.021713176 | 10.70108718 | 8.12E-08 | 1.62E-06 |
| CXCL11_261361 | 4.712702755 | 12.17691208 | 8.78E-08 | 1.66E-06 |
| IDO1_268369 | 2.77746775 | 10.21297062 | 1.32E-07 | 2.38E-06 |
| FOXM1_10551166 | 3.511025369 | 7.302016812 | 3.85E-07 | 6.66E-06 |
| LAMP3_12361344 | 2.218476016 | 9.184121563 | 4.93E-07 | 8.17E-06 |
| IFIT3_72174 | 2.826535538 | 12.01665835 | 6.87E-07 | 1.07E-05 |
| MAD2L1_115221 | 1.749064486 | 8.034765012 | 7.01E-07 | 1.07E-05 |
| CD38_519628 | 1.940842458 | 9.824900589 | 7.36E-07 | 1.08E-05 |
| CCR1_54149 | 1.473037432 | 10.11920316 | 8.34E-07 | 1.19E-05 |

| | | | | |
|---|---|---|---|---|
| GZMB_581688 | 1.898001877 | 10.41173908 | 8.96E-07 | 1.23E-05 |
| LAG3_13111419 | 2.386185632 | 7.994487875 | 1.01E-06 | 1.34E-05 |
| TCF7_677799 | -1.154491364 | 6.98289475 | 1.15E-06 | 1.48E-05 |
| IFI44L_12771376 | 2.551619364 | 11.04872147 | 1.20E-06 | 1.49E-05 |
| TOP2A_27522855 | 2.412862908 | 9.724530736 | 1.28E-06 | 1.55E-05 |
| KLF2_9111017 | -1.146655278 | 10.32922348 | 1.82E-06 | 2.13E-05 |
| MX1_232336 | 2.598359407 | 11.58716187 | 2.53E-06 | 2.88E-05 |
| CXCL9_149250 | 2.475393657 | 11.64602422 | 3.36E-06 | 3.71E-05 |
| CD276_12011310 | 1.404468545 | 6.909158714 | 3.83E-06 | 4.12E-05 |
| STAT1_18871996 | 1.341291438 | 12.54396256 | 4.11E-06 | 4.31E-05 |
| IFI35_419526 | 1.590766479 | 9.999010988 | 4.93E-06 | 5.03E-05 |
| IFIH1_20172123 | 1.978276916 | 9.64184768 | 5.53E-06 | 5.50E-05 |
| CCL18_198296 | 2.559340347 | 11.50058854 | 6.63E-06 | 6.43E-05 |
| KLRB1_177284 | -1.356445562 | 10.32648685 | 7.21E-06 | 6.84E-05 |
| CXCR4_100208 | -1.262015044 | 12.69186025 | 8.67E-06 | 8.03E-05 |
| GBP1_771872 | 1.624043959 | 12.95069926 | 1.21E-05 | 0.000109887 |
| CEACAM8_745847 | -2.38182276 | 6.835412175 | 1.51E-05 | 0.000133479 |
| IFIT2_124224 | 2.601872644 | 12.64896588 | 1.80E-05 | 0.000155644 |
| PDCD1LG2_315423 | 1.435337704 | 9.765019917 | 1.94E-05 | 0.000164473 |
| CXCL10_354459 | 2.921361689 | 12.20854003 | 2.10E-05 | 0.000173779 |
| IFIT1_158259 | 3.138880653 | 12.7962216 | 2.30E-05 | 0.000186992 |
| BCL6_21502257 | -1.128692675 | 11.38395943 | 2.37E-05 | 0.000188627 |
| CD69_195303 | -1.167440855 | 10.29551815 | 2.49E-05 | 0.000194401 |
| PTGS2_14761583 | -1.787690656 | 9.487046641 | 3.11E-05 | 0.000229188 |
| CD226_9021006 | -1.055303117 | 8.204262834 | 3.51E-05 | 0.000249595 |
| C1QB_111199 | 1.318021235 | 14.14106331 | 5.40E-05 | 0.000370819 |
| CXCL13_202307 | 2.634779914 | 10.17220128 | 5.53E-05 | 0.000372978 |
| CCNB2_9861095 | 2.451586323 | 8.610014511 | 6.34E-05 | 0.000420281 |
| BRCA2_98179922 | 1.408177984 | 6.624940626 | 6.59E-05 | 0.000429787 |
| MKI67_581686 | 1.830582251 | 9.016084457 | 8.31E-05 | 0.000533209 |

| | | | | |
|---|---|---|---|---|
| BCL2_10401144 | -1.054459851 | 7.084554684 | 9.27E-05 | 0.000576492 |
| DDX58_540643 | 1.637092216 | 11.5957064 | 0.000122508 | 0.000750128 |
| KLRG1_410518 | -1.049817337 | 6.871878081 | 0.000129441 | 0.000780569 |
| SNAI2_722828 | 1.272531289 | 9.779931728 | 0.000139876 | 0.000830906 |
| POU2AF1_230337 | 1.981866553 | 9.738343726 | 0.000172607 | 0.000967573 |
| XAGE1B_469547 | 2.99835029 | 4.93494266 | 0.000177305 | 0.000980102 |
| C1QA_67171 | 1.197339812 | 12.61310409 | 0.000183576 | 0.000989574 |
| PTPRC_710817 | -1.011649066 | 9.527123762 | 0.000183991 | 0.000989574 |
| CCL17_288394 | -1.834523807 | 6.992710035 | 0.0002361 | 0.001220359 |
| CD1C_12531357 | -2.519073627 | 6.111702351 | 0.000306038 | 0.001541811 |
| MPO_15121620 | -1.908031327 | 8.211592408 | 0.00032134 | 0.001598665 |
| CCR5_85193 | 1.040859769 | 9.653446555 | 0.00037676 | 0.001851239 |
| CD83_480580 | -1.053864486 | 8.523628945 | 0.000393979 | 0.001889203 |
| CRTAM_312417 | -1.623243078 | 5.490123515 | 0.000404446 | 0.001916303 |
| HERC6_17021806 | 1.846150693 | 8.504611085 | 0.000412365 | 0.001930837 |
| BST2_218322 | 1.11377782 | 14.61484839 | 0.000474995 | 0.002148274 |
| HLA-DQB2_142243 | -2.208546554 | 2.6772892 | 0.000635872 | 0.00275705 |
| CMKLR1_36143 | 3.089416269 | 4.095184302 | 0.000921508 | 0.003860634 |
| ISG20_642750 | 1.288727823 | 10.51232195 | 0.001035118 | 0.004203845 |
| TNFSF18_128228 | 2.048786407 | 5.115450907 | 0.001219778 | 0.004854718 |
| CD163_21422245 | 1.190748679 | 13.00811752 | 0.001277279 | 0.005033238 |
| CYBB_14221529 | 1.034200312 | 11.76129988 | 0.001416071 | 0.005525454 |
| IFITM1_359459 | 1.094499983 | 14.73592207 | 0.001527366 | 0.005845111 |
| RORC_12011307 | -1.232621407 | 6.368975553 | 0.001648956 | 0.00625033 |
| TNFRSF9_894998 | -1.557976838 | 6.277732459 | 0.002060615 | 0.007593748 |
| IL2_366451 | -1.787299469 | 2.137968085 | 0.002118414 | 0.007735127 |
| IRF4_786895 | 1.2991475 | 7.589669323 | 0.002433884 | 0.008497246 |
| IL21_368450 | 2.713025334 | 3.179107785 | 0.002892483 | 0.009924208 |
| FCGR1A_547652 | 1.358837352 | 8.984718333 | 0.003056301 | 0.010396649 |
| SLAMF7_9161020 | 1.178583741 | 10.97955754 | 0.00366687 | 0.012263986 |

| | | | | |
|---|---|---|---|---|
| GZMA_165265 | 1.05539306 | 10.51173829 | 0.004314939 | 0.014311214 |
| CCR6_271363 | -1.108756703 | 5.421780719 | 0.004727498 | 0.015422493 |
| ARG1_174278 | -2.016630732 | 7.856291723 | 0.005246505 | 0.016976497 |
| IL10_491598 | 1.240451265 | 6.779053894 | 0.005949936 | 0.018944598 |
| TNFRSF17_254359 | 1.607351136 | 7.694337923 | 0.006172838 | 0.019498329 |
| MAGEC2_249358 | -2.203350303 | -1.572033969 | 0.007805425 | 0.024081854 |
| CXCR5_153252 | -1.570791614 | 3.754056017 | 0.009131323 | 0.027955897 |
| KRT5_10631165 | 2.093456369 | 10.18852794 | 0.009780171 | 0.029488696 |
| KRT7_440543 | 1.017265847 | 12.56930876 | 0.012411449 | 0.035795337 |
| GAGE10_137250 | -2.028791387 | 1.82618068 | 0.018144659 | 0.049462838 |

Significance calculated using binomial test generalized to overdispersed counts, fdr corrected.

Supplementary Table 4. Antibodies and staining conditions

| Antibody | Supplier | Product Number | Clone | Dilution | Pretreatment | Staining platform | Detection system |
|---|---|---|---|---|---|---|---|
| CD3 | Novocastra | NCL-L-CD3-565 | LN10 | 1:100 | H2(20)100 | Bond III | Bond Polymer Refine Red Detection (DS9390) |
| CD4 | Novocastra | CD4-368-L-CE | 4B12 | 1:80 | 30min ER2 95°C | Bond III | Bond Polymer Refine Red Detection (DS9390) |
| CD8 | Novocastra | NCL-L-CD8-4B11 | 4B11 | 1:40 | H2(30)95 | Bond III | Bond Polymer Refine Red Detection (DS9390) |
| CD15 | Cellmarque | 115M | MMA | 1:25 | H1(20)100 | Bond III | Bond Polymer Refine Red Detection (DS9390) |
| CD20 | Agilent | M0755 | L26 | 1:600 | H2(20)95 | Bond III | Bond Polymer Refine Red Detection (DS9390) |
| CD68 | Agilent | M0876 | PG-M1 | 1:100 | E1(5) | Bond III | Bond Polymer Refine Red Detection (DS9390) |
| CD123 | Novocastra | CD123-L-CE | BR4MS | 1:20 | 20min ER2 95°C | Bond III | Bond Polymer Refine Red Detection (DS9390) |
| CD163 | Novocastra | NCL-L-CD163 | 10D6 | 1:200 | H1(20)100 | Bond III | Bond Polymer Refine Red Detection (DS9390) |
| C3d | Dako | A0063 | polyclonal | 1:700 | E1(10) | Bond III | Bond Polymer Refine Red Detection (DS9390) |
| C5b-9 | Lifespan Biosciences | | aE11 | 1:50 | Enzyme 1 (5) | Benchmark GX | OptiView DAB (Bestellnummer: 760-700) |
| Ki67 | Agilent | M7240 | MIB-1 | 1:50 | H2(20)95 | Bond III | Bond Polymer Refine Red Detection (DS9390) |
| MPO | Agilent | A0398 | polyclonal | 1:8000 | H2(20)95 | Bond III | Bond Polymer Refine Red Detection (DS9390) |
| p53 | Agilent | M7001 | D07 | 1:1200 | H1(20)100 | Bond III | Bond Polymer Refine Red Detection (DS9390) |
| PD1 | Roche Ventana | 760-4895 | NAT105 | no dilution | CC1 40min | Benchmark GX | OptiView DAB (Bestellnummer: 760-700) |
| SARS-CoV-2 | Rockland | 200-401-A50 | Anti-SARS-CoV Nucleocapsid | 1:6400 | H2(20)95 | Bond III | Bond Polymer Refine Red Detection (DS9390) |

Supplementary Table 5. Histopathology

| Case No. | Severity of histological changes in lungs [1] | DAD stage [2] | Intraalveolar edema [3] | Intraalveolar hemorrhage [3] | Neutrophils [4] | SARS-COV-2 genomes / 10^6 RNaseP copies | SARS-CoV-2 IHC [4] |
|---|---|---|---|---|---|---|---|
| C12 | 1 | - | 1 | 1 | 1 | 673 | 1 |
| | 2 | 1 | 1 | 1 | 2 | 2'871 | 2 |
| | 2 | 1 | 1 | 1 | 2 | 116'310 | 3 |
| C13 | 3 | 1 and 2 | 1 | 1 | 2 | 38 | 0 |
| | 3 | 1 and 2 | 1 | 1 | 2 | 310 | 0 |
| | 3 | 1 and 2 | 1 | 1 | 2 | 225 | 0 |
| C16 | 1 | 1 | 1 | 0 | 1 | 91 | 0 |
| | 1 | 1 and 2 | 1 | 0 | 1 | 20 | 0 |
| C17 | 1 | 2 | 1 | 1 | 1 | 12 | 0 |
| | 2 | 1 and 2 | 1 | 1 | 2 | 25 | 0 |
| C19 | 1 | 1 | 1 | 1 | 1 | 7'603 | 2 |
| | 1 | 1 | 0 | 1 | 1 | 18'867 | 2 |
| C20 | 3 | 1 and 2 | 1 | 1 | 2 | 145 | 0 |
| | 2 | 1 and 2 | 1 | 1 | 2 | 3'205 | 1 |
| | 3 | 1 and 2 | 1 | 1 | 2 | 32 | 0 |
| C21 | 3 | 2 | 0 | 0 | 2 | 119 | 0 |
| | 3 | 2 | 0 | 0 | 1 | 53 | 0 |
| | 2 | 2 | 0 | 0 | 1 | 119 | 0 |
| C15 | 2 | 1 | 1 | 0 | 2 | 91'186 | 3 |
| | 2 | 1 | 1 | 0 | 1 | 2'993 | 2 |
| C1 | 1 | - | 1 | 0 | 1 | 0.4 | 0 |
| | 1 | - | 1 | 0 | 1 | 0.0 | 0 |
| | 1 | - | 1 | 0 | 1 | 0.2 | 0 |
| C3 | 2 | - | 0 | 0 | 3 | 14'311 | n.d. |
| | 2 | - | 1 | 1 | 3 | 249'937 | 3 |
| | 2 | - | 0 | 0 | 3 | 157'042 | 2 |
| C4 | 1 | - | 0 | 1 | 1 | 314'728 | 3 |
| | 2 | - | 1 | 1 | 1 | 245'331 | 3 |
| | 2 | 1 | 1 | 1 | 1 | 187'540 | 3 |
| C5 | 2 | - | 0 | 0 | 1 | 8 | 0 |
| | 2 | 1 and 2 | 0 | 0 | 1 | 180 | 0 |
| | 2 | 1 and 2 | 0 | 1 | 2 | 82 | 0 |
| C6 | 1 | - | 0 | 0 | 1 | 10'451 | 1 |
| | 1 | - | 0 | 0 | 1 | 118'620 | 3 |

| | | | | | | | |
|---|---|---|---|---|---|---|---|
| | 1 | - | 1 | 0 | 1 | 114'407 | 3 |
| C7 | 1 | 1 and 2 | 0 | 0 | 1 | 12'939 | 3 |
| | 1 | 1 and 2 | 0 | 0 | 1 | 1'443 | 1 |
| | 1 | 1 and 2 | 0 | 1 | 2 | 16'534 | 2 |
| C8 | 1 | - | 0 | 0 | 1 | 17'598 | n.d. |
| | 3 | - | 1 | 0 | 3 | 316 | n.d. |
| | 1 | - | 0 | 0 | 1 | 139 | n.d. |
| C9 | 1 | - | 0 | 0 | 1 | 64'242 | n.d. |
| | 1 | - | 0 | 0 | 1 | 85'137 | n.d. |
| | 1 | - | 1 | 0 | 1 | 29'487 | n.d. |

At least two different tissue blocks from different areas of the lungs were evaluated for each case.
[1] 1 = slight to moderate changes; 2 = moderate changes; 3 = severe changes
[2] 1 = exudative; 2 = proliferative / organizing; 3 = fibrotic
[3] 1 = yes; 0 = no
[4] 1 = very few or few; 2 = moderate; 3 = numerous
