# Supplementary material for: Two distinct immunopathological profiles in autopsy lungs of COVID-19
Source: Nat Commun. 2020 Oct 8;11:5086. doi: 10.1038/s41467-020-18854-2 (PMC7546638; doi:10.1038/s41467-020-18854-2)
Supplement: Supplementary file 3 — Reporting Summary [file 41467_2020_18854_MOESM3_ESM.pdf]

nature research

Corresponding author(s): Kirsten D. Mertz

Last updated by author(s): Sep 9, 2020

Reporting Summary

Nature Research wishes to improve the reproducibility of the work that we publish. This form provides structure for consistency and transparency in reporting. For further information on Nature Research policies, see our Editorial Policies and the Editorial Policy Checklist.

Statistics

For all statistical analyses, confirm that the following items are present in the figure legend, table legend, main text, or Methods section.

n/a	Confirmed	
☐	☒	The exact sample size (*n*) for each experimental group/condition, given as a discrete number and unit of measurement
☐	☒	A statement on whether measurements were taken from distinct samples or whether the same sample was measured repeatedly
☐	☒	The statistical test(s) used AND whether they are one- or two-sided<br>*Only common tests should be described solely by name; describe more complex techniques in the Methods section.*
☒	☐	A description of all covariates tested
☐	☒	A description of any assumptions or corrections, such as tests of normality and adjustment for multiple comparisons
☐	☒	A full description of the statistical parameters including central tendency (e.g. means) or other basic estimates (e.g. regression coefficient) AND variation (e.g. standard deviation) or associated estimates of uncertainty (e.g. confidence intervals)
☐	☒	For null hypothesis testing, the test statistic (e.g. F, t, r) with confidence intervals, effect sizes, degrees of freedom and P value noted<br>*Give P values as exact values whenever suitable.*
☒	☐	For Bayesian analysis, information on the choice of priors and Markov chain Monte Carlo settings
☒	☐	For hierarchical and complex designs, identification of the appropriate level for tests and full reporting of outcomes
☐	☒	Estimates of effect sizes (e.g. Cohen's *d*, Pearson's *r*), indicating how they were calculated

Our web collection on statistics for biologists contains articles on many of the points above.

Software and code

Policy information about availability of computer code

Data collection	Completely disclosed in Methods.<br>Commercial software / systems were used to process the raw sequencing data (OIRRA, HALO software and software packages, all described in the Methods section), and data post-processing and representation were performed with standard R functions (all listed in the Methods section). In brief, our study does not rely on any custom algorithms or software that are central to the research but not yet described in published literature.<br>De-multiplexing and gene expression level quantification were performed with the standard setting of the ImmuneResponseRNA plugin (version 5.12.0.1) within the Torrent Suite (version 5.12.1), provided as part of the OIRRA by Thermo Fisher Scientific, Waltham, MA, USA.<br>All the analyses and graphical representations were performed using the R statistical environment software21 and the following packages: ggplot2, circlize, ComplexHeatmap, ggfortify, reshape2 and factoextra. Correlation between transcripts and viral counts was performed using Pearson's correlation. Association between continuous and categorical data were tested using Wilcoxon rank sum test.<br>CLC genomics workbench (version 20.0.3, Qiagen, Hilden, Germany) was used in combination with the microbial genomics module (version 20.0.1, Qiagen, Hilden, Germany).<br>HALO Software version: 3.0.311.167
Data analysis	Commercial software / systems were used to process the raw sequencing data (OIRRA, HALO software and software packages, all described in the Methods section), and data post-processing and representation were performed with standard R functions (all listed in the Methods section). In brief, our study does not rely on any custom algorithms or software that are central to the research but not yet described in published literature.<br>De-multiplexing and gene expression level quantification were performed with the standard setting of the ImmuneResponseRNA plugin (version 5.12.0.1) within the Torrent Suite (version 5.12.1), provided as part of the OIRRA by Thermo Fisher Scientific, Waltham, MA, USA.<br>All the analyses and graphical representations were performed using the R statistical environment software21 and the following packages: ggplot2, circlize, ComplexHeatmap, ggfortify, reshape2 and factoextra. Correlation between transcripts and viral counts was performed using

Pearson's correlation. Association between continuous and categorical data were tested using Wilcoxon rank sum test.
CLC genomics workbench (version 20.0.3, Qiagen, Hilden, Germany) was used in combination with the microbial genomics module (version 20.0.1, Qiagen, Hilden, Germany).
HALO Software version: 3.0.311.167

For manuscripts utilizing custom algorithms or software that are central to the research but not yet described in published literature, software must be made available to editors and reviewers. We strongly encourage code deposition in a community repository (e.g. GitHub). See the Nature Research guidelines for submitting code & software for further information.

Data

Policy information about availability of data

All manuscripts must include a data availability statement. This statement should provide the following information, where applicable:

- Accession codes, unique identifiers, or web links for publicly available datasets
- A list of figures that have associated raw data
- A description of any restrictions on data availability

The datasets generated and analysed during this study can be accessed in GEO (accession number GSE151764) and are available from the corresponding author upon request. The GEO dataset with the accession number GSE151764 is now publicly available:
https://www.ncbi.nlm.nih.gov/gds/?term=GSE151764[Accession]
The hyperlink for the NCBI Reference Sequence Database (https://www.ncbi.nlm.nih.gov/refseq/) is provided in the Methods section.
List of figures that have associated raw data: Figure 1a, raw data shown in Supplementary Table 3.
A complete data availability statement is provided in the manuscript.

Field-specific reporting

Please select the one below that is the best fit for your research. If you are not sure, read the appropriate sections before making your selection.

☒ Life sciences ☐ Behavioural & social sciences ☐ Ecological, evolutionary & environmental sciences

For a reference copy of the document with all sections, see nature.com/documents/nr-reporting-summary-flat.pdf

Life sciences study design

All studies must disclose on these points even when the disclosure is negative.

Sample size	All COVID-19 lung autopsy tissues obtained from two institutions (Cantonal Hospital Baselland, Liestal, and University Hospital Basel, Switzerland) between March 9th and April 14th 2020 were included into the study. In spite of the limited sample size (n=34), unsupervised analysis resulted in discovery of two cateogries with highly significant differences based on ISG expression and immune cell infiltration of tissues. Therefore we consider the sample size sufficient for the conclusions made in this study.
Data exclusions	For comparison of ISG-high and ISG-low patterns, 3/34 COVID-19 lung samples were not taken into account since they did not fall into either of these categories. The exclusion criteria were not pre-established. When we performed our analysis, we realized that 3 samples were outliers as they did not cluster with the other COVID-9 lung samples. Therefore we mention that these 3 outlier samples cluster in the group of normal samples, and, as mentioned in the Results and in Figure Legend 1, these 3 samples were not taken into account when comparing the lung samples of the ISGhigh and ISGlow groups.
Replication	Data were not replicated with an independent COVID-19 autopsy cohort, because none is available at this point in time.
Randomization	All COVID-19 autopsies from our institutions were taken into account without randomization.
Blinding	Histology was evaluated by at least three board-certified pathologists (VK, VZ, NW) in a blinded fashion. Blinded quantification of immunohistochemistry was done in an automated fashion. For evaluation of histology, data collection and analysis, all pathologists were completely blinded to group allocation.

Reporting for specific materials, systems and methods

We require information from authors about some types of materials, experimental systems and methods used in many studies. Here, indicate whether each material, system or method listed is relevant to your study. If you are not sure if a list item applies to your research, read the appropriate section before selecting a response.

Materials & experimental systems

n/a	Involved in the study	
☐	☒	Antibodies
☒	☐	Eukaryotic cell lines
☒	☐	Palaeontology and archaeology
☒	☐	Animals and other organisms
☐	☒	Human research participants
☒	☐	Clinical data
☒	☐	Dual use research of concern

Methods

n/a	Involved in the study	
☒	☐	ChIP-seq
☒	☐	Flow cytometry
☒	☐	MRI-based neuroimaging

Antibodies

Antibodies used	Completely disclosed in Supplementary Table 4.
Validation	Antibody stainings are part of the diagnostic routine at our institution, i.e. negative and positive controls have been established and validated on tonsil sections for all antibodies used, including isotype controls for monoclonals. All validation protocols are documented in accreditation protocol ISO 15189:2012 of our institution and are available upon request. This has been mentioned in the Methods section.

Human research participants

Policy information about studies involving human research participants

Population characteristics	Fully disclosed in Table 1 and Supplementary Table 1.
Recruitment	All COVID-19 autopsy cases obtained in our two institutions (Cantonal Hospital Baselland, Liestal, and University Hospital Basel, Switzerland) between March 9th and April 14th 2020 were included in our study.<br>As mentioned in the Discussion, the gene signature for cytotoxic T cells and the specific cytokines analyzed in Figure 5 were chosen based on the published literature, linking the expression of specific genes with severe COVID-19. With the limited number of genes on our expression array, an unbiased analysis was not deemed feasible. We recognize and specifically discuss that a pre-defined set of genes to define functional modules may have introduced an experimental bias. To avoid a potential bias in our sampling of lung specimens, we analyzed at least two samples of each patient from different lung areas.
Ethics oversight	Ethics approval was obtained from the Ethics Committee of Northwestern and Central Switzerland (Project-ID 2020-00629). For all patients, either personal and / or family consent was obtained for autopsy and sample collection.

Note that full information on the approval of the study protocol must also be provided in the manuscript.
